# Would victims blame victims? Effects of ostracism, sexual objectification, and empathy on victim blaming

**DOI:** 10.3389/fpsyg.2022.912698

**Published:** 2022-08-01

**Authors:** Maayan Dvir, Maayan Nagar

**Affiliations:** ^1^The Center for Psychobiological Research, Department of Psychology, Max Stern Yezreel Valley College, Yezreel Valley, Israel; ^2^School of Criminology, Faculty of Law, Haifa University, Haifa, Israel

**Keywords:** victim blame, ostracism, social exclusion, empathy, sexual objectification, eye gaze, objectifying gaze

## Abstract

In the current research, we examined whether ostracism and sexual objectification affect the tendency to blame the victim of sexual harassment. Previous research concerning victim blame examined the attribution of blame considering the characteristics of the victim, the perpetrator, and the relation between them. However, no research to date examined whether situational factors of the perceiver can affect their perception and judgment of blame. We propose that sexual objectification and ostracism may elicit empathy toward the victim, and in turn, reduce victim blame. In two experimental studies, women were instructed to imagine interacting with a videotaped man who either gazed at their body (objectification), away from them (ostracism), or at their face (treated well). Then, they were asked to read a newspaper article (study 1) or watch a video (study 2) portraying encounters in which the man's sexual advances continued after the woman expressed discomfort and lack of interest. In study 1, we found that sexually objectified women attributed less blame to the woman compared with the women who were treated well, with ostracized women falling in between and marginally different from both. In study 2, using mediation analysis we found an indirect effect such that sexually objectified women experienced greater empathy toward the victim, which was associated with reduced attribution of blame. It appears that greater similarity between the situation of the perceiver and the situation of the victim elicits greater empathy. This adds to the previous knowledge that personality similarities result in higher empathy.

Sexual objectification such as unwanted sexual looks or gestures, texts and calls of sexual nature, attempted sexual assaults, and others, is extremely common, with women are two times as much likely to be the victims (81% of women compared with 43% of men; Stop Street Harassment., 2018). During 2017, the #MeToo movement raised awareness to sexual harassment and encouraged victims to report their experiences with sexual harassment, rapidly gaining popularity and spreading worldwide. Surprisingly, even then, victims of sexual harassment and assault were frequently accused of being responsible, at least to some extent, for the incident (e.g., Lucarini et al., 2020). Although men are more likely to engage in victim blaming toward a female victim, studies have shown that women engage in victim blaming as well (e.g., Culda et al., 2018). Scholars argue that women who engage in victim blaming do so as a means of self-defense (Culda et al., 2018). Once you believe that one is responsible for her own destiny, it is easier to believe that you will not find yourself in a similar situation. Victim blaming leaves the victim hurt and alone, ostracized, and excluded by others. Past research has yet to examine how the situation in which women are in may affect the perception of these events and their attribute of blame toward a victim of sexual harassment. The aim of this research is to explore whether being the victim of sexual objectification or ostracism affects women's attribution of blame to other victims.

Ostracism is defined as being ignored and excluded (Williams, 2009). Once an individual detects signs of ostracism, feelings of pain and negative affect rise, in addition to threat to four fundamental needs: the need to belong and possess social relations, the need to be in control of one's social situation, the need to maintain positive self-esteem, and the need to believe that one's existence has meaning (Williams, 2009). The aim to restore those threatened needs may alter the ostracized individual's perceptions of social situations. Whereas, most research focused on complete ostracism, when one is completely ignored and excluded, some research also explored partial ostracism, in which one is ignored and excluded intermittently—such as being kept out of the loop on a certain topic while still being included in the conversation (Jones et al., 2009; Iannone et al., 2018) or receiving some attention but less so than what would be expected as fair inclusion (Williams, 2007). Importantly, recent research demonstrated that women experience sexual objectification as a form of partial ostracism and as a result experience threat to the same fundamental needs (Dvir et al., 2021). Sexually objectified women realize that their body is the focus of attention, but simultaneously feel that their thoughts and feelings are disregarded, and thus feel ostracized.

Sexual objectification occurs when one is treated as if her body and sexual function represent her as a whole, as if she is merely a body that exists for the use and pleasure of others (Bartky, 1990; Fredrickson and Roberts, 1997). Sexual objectification of women is a significant part of the socialization of girls and women in the western societies, is expressed in the way women are portrayed in media, and is a part of the daily experience of women in interpersonal interactions when they are treated as if their bodies represent them (Fredrickson and Roberts, 1997; Szillis and Stahlberg, 2007; Holland et al., 2017). The eye gaze alone has been known to signal sexual objectification when one is leering at a woman's body (i.e., the objectifying gaze). Various research demonstrated the harmful effects of sexual objectification on women's mood and well-being, self-perception, self-presentation and cognitive performance (Moradi and Huang, 2008; Saguy et al., 2010; Gervais et al., 2011, 2013; Guizzo and Cadinu, 2017).

Empathy is the human capacity to understand, share, and respond to the emotions of others. It is a complex process that is achieved by two levels: on the basic level, empathy is achieved through direct perception of another person's behavior, implying one's automatic feelings toward the other (emotional empathy); on the higher level, empathy is achieved through cognition, involving psychological understanding, inference from social cues, communication, and perspective taking (cognitive empathy) (Decety, 2005; Smith, 2006; Fuchs, 2017). These levels mutually influence one another. It is argued that empathy is a psychological process of involvement, which evokes one's emotional response. This emotional response is expressed as one feels what is appropriate for another person's situation, not one's own (Hoffman, 2000).

Since we, as humans, are equipped with a mechanism to enhance social connection, one might wonder about the effects of social exclusion on our empathic mechanism. In other words, how this mechanism of social bonding will work when our social ties are torn apart. On the one hand, being ostracized (fully or partially) may illicit one's emotional responses, making them hypersensitive to others, while trying harder to be a part of a group and increase social interactions with others. On the other hand, ostracism may deplete one's cognitive and affective resources, leading to lower capacity to react empathically to another person's suffering. Research, thus far, has supported both directions. Most of the studies have supported the latter response showing that ostracized individuals become more aggressive toward others (Twenge et al., 2001; Twenge and Campbell, 2003; Buckley et al., 2004), are less willing to help and present less prosocial behavior (Coyne et al., 2011; van Bommel et al., 2016; Kothgassner et al., 2017), and showed less empathy for another person's suffering (DeWall and Baumeister, 2006). However, some studies have supported the former direction, indicating that ostracized individuals showed greater perspective taking (e.g., were able to instruct a blindfolded other through a maze) compared with non-ostracized individuals (Knowles, 2014), and greater sensitivity to social cues (Pickett et al., 2004; Nordgren et al., 2011). In addition, studies found that watching another person being ostracized triggers an automatic empathic response to ostracism, causing the observer to react as if they are being ostracized themselves. This effect was magnified when participants were instructed to empathize with the ostracized individual (Wesselmann et al., 2009). Interestingly, ostracism does not elicit empathy toward ostracized women if they are portrayed in a sexually objectified manner. In a study, participants were ostracized, and then watched a sexually objectified (vs. personalized) woman being ostracized (Cogoni et al., 2021). Then, they rated how they felt during the task and how the target felt (very negative to very positive). Results indicated that individuals' rating of the target's feelings was less congruent with their own feelings when the target was sexually objectified (compared with not objectified). Following ostracism, individuals were less able to empathize with a sexually objectified woman. Other studies have not found significant effects of ostracism on empathy (Bass et al., 2014; Kandaurova and Lee, 2019).

Unlike ostracism, the effects of sexual objectification on empathy have not been examined thus far. It is important to note that only recently researchers started to examine the causal effects of sexual objectification. Reason being that sexual objectification manipulations are elaborate and expensive, require lab setting and trained confederates (e.g., Saguy et al., 2010; Gervais et al., 2011; Dvir et al., 2021). However, leaning on the empathy literature that illustrated the importance of perceiving the other as similar to the self in eliciting empathy (Davis, 1994; Batson et al., 2005), we propose that the more people perceive the situation others are in as similar to their own, the better they can relate and empathize with them. It is therefore interesting to examine how empathy, whether induced or reduced by sexual objectification and ostracism, will affect victim blaming.

The literature on victim blame have focused on characteristics of the victim and her behavior, characteristics of the perpetrator and his relation to the victim, and characteristics of the situation they were in. Mainly, the attribution of the blame to the victim is more likely when alcohol or drugs are present (Wild et al., 1998; Hayes-Smith and Levett, 2010); when the victim was wearing more revealing clothes (Whatley, 2005; Loughnan et al., 2013); when the victim did not attempt to resist the assault (Krulewitz, 1981); and in cases when the victim and the perpetrator had previous association with one another (acquaintance assault; e.g., Grubb and Harrower, 2008). In addition, little research has examined the effects of empathy for the victim on the attribution of blame. In general, empathy for the victim correlated with less victim blaming (Diehl et al., 2014; Gravelin et al., 2019; Bongiorno et al., 2020). No study to date has examined how situational factors of the observer (e.g., being ostracized) affect the tendency to blame the victim.

In two studies, we aimed to examine the interplay between ostracism, sexual objectification (partial ostracism), empathy, and victim blame among women. In study 1, we examined whether ostracized and objectified women would attribute less blame to a sexually harassed woman in a newspaper article. In study 2, we aimed to examine empathy as a potential mechanism that attenuates the effect of ostracism and sexual objectification on victim blame.

## Study 1

### Methods

#### Participants and design

In total, 146 women participated in the study virtually from their personal computers (*M*_*age*_ = 25.98, *SD* = 3.91; Range_Age_ = 18–39). Most participants identified as heterosexual (96.6%). An *a-priori* power analysis to achieve 80% power (α = 0.05; *partial* η^2^ = 0.07) determined a desired sample size of 138 participants. Link to the study was distributed on social media with a post inviting to volunteer for a study about interpretation of social situations. Participants were randomly assigned to one of three conditions: ostracism, sexual objectification, and control.

#### Procedure

After reading a short description of the study and indicating their informed consent, participants were asked to practice their mental visualization skills by imagining they are interacting with the person who will appear in the upcoming video. They were asked to imagine that they just met and interact with the person for the first time. Participants were asked to mentally visualize the situation to the best of their ability, by imagining the topic of the conversation, the characteristics of the situation, and the identity of the person. The participants watched a 2-minutes video portraying a man where the man's eye gaze was manipulated (Dvir et al., 2021). Participants were randomly assigned to be either ostracized—the man's eye gaze alternated between directly at the participants face and *away* to the side; sexually objectified—the man's eye gaze alternated between directly at the participants face and down to her *body*; or treated well (control condition)—the man's eye gaze was directly at the participants *face* for the whole time. After watching the video, the participants described what they mentally visualized during the exercise, and completed objectification-related questionnaires.

Then, the participants read a bogus newspaper article describing an encounter between two students, a man and a woman, from the woman's perspective (see Supplementary Material). The article purposefully described a situation that is regarded in the media as being in the “gray area” to allow for different interpretations. Throughout the article, the man's sexual advances continue and become fiercer. The woman describes being confused and reluctant at first; gradually, she becomes upset, expressing her discomfort and lack of interest. Because the man persisted, the woman eventually left. After reading the article, participants answered questions regarding their interpretation of the event described in the article and victim blaming.

At last, participants completed manipulation and attention checks, indicated whether they encountered technical issues, and completed a demographic background questionnaire.

#### Measures

Unless otherwise is specified, all the measures^1^ were on a 7-point scale ranging from not at *all* (1) to *extremely* (7).

##### Objectification-related measures

###### Sexual objectification

Participants rated their agreement with the statements “I felt objectified” and “I felt sexually objectified” during the interaction (2 items; α= 0.90).

###### Self-objectification

Participants indicated their agreement with statements on the State Self-Objectification Scale regarding their feelings while imagining the interaction (Saguy et al., 2010); 3 items; e.g. “I felt as though I am more of a body than a person”; α = 0.85.

##### Victim blame

Participants indicated the extent to which they believe that the woman was at fault for the incident, that the woman's behavior elicited the man's actions, and the extent to which the woman “asked for it” (3 items; e.g., “To what extent the woman in the article is at fault for what happened?”; α = 0.80).

##### Manipulation and attention checks

###### Eye gaze direction

Participants indicated the direction of the person in the video's eye gaze (checked all that may apply: upward, downward, forward, to the side, and other).

###### Subject of article

Participants were asked to indicate the subject of the article: meeting between friends from the man's/woman's perspective, blind date from the man's/woman's perspective, or business meeting from the employer's/employee's perspective.

#### Statistical analysis

To examine the effects of the condition (i.e., *away*- ostracism, *body*- sexual objectification, and *face*- control) on the outcome variables a series of one-way ANOVA was conducted, unless stated otherwise.

### Results and discussion

### Preliminary analysis

#### Manipulation checks

##### Eye gaze direction

A chi-square test of independence was performed to examine the relation between the video conditions and the participant's perception of the man's eye gaze direction. The relation was significant, $\chi_{\left(4\right)}^{2}$ = 227.16, *p* < 0.001, indicating that the majority of the participants in each condition correctly identified the direction of the man's eye gaze: Ostracism (side eye gaze; 90.2%), sexual objectification (down eye gaze; 90.9%), and control (direct eye gaze; 94%).

##### Subject of article

Most participants reported reading an article about a meeting between friends from the woman's perspective (91.8%).

#### Process check

We used process checks to examine whether the manipulation was not only noticeable but also elicited the process intended (e.g., downward eye gaze to the body is interpreted as sexual objectification). Participants in the *sexual objectification (body)* condition felt more sexually objectified as compared with both the *control (face)* and *ostracism (away)* conditions, *F*_(2, 142)_ = 160.32, *p* < 0.001, *partial* η^2^= 0.69 (LSD simple effects < 0.001). In addition, participants in the *sexual objectification* condition reported higher self-objectification as compared with the *control* and *ostracism* conditions, *F*_(2, 143)_ = 120.98, *p* < 0.001, *partial* η^2^= 0.63 (LSD simple effect *p*s < 0.001). For means and SDs see Table 1.

**Table 1:** Means and standard deviations of sexual and self-objectification as a function of condition (face, away, body) in Study 1 and Study 2.

	**Study 1**		**Study 2**	
	**Sexual objectification**	**Self-objectification**	**Sexual objectification**	**Self-objectification**
Face	1.17 ± 0.50	1.46 ± 0.76	1.60 ± 0.80	2.05 ± 1.02
Away	1.54 ± 0.93	1.62 ± 0.98	1.98 ± 1.16	2.42 ± 1.40
Body	5.47 ± 2.00	4.90 ± 1.74	3.21 ± 2.17	2.80 ± 1.62

### Main analysis

#### Victim blame

Analysis of variance revealed a significant effect for condition, *F*_(2, 143)_ = 7.78, *p* = 0.001, *partial* η^2^= 0.10. Participants in the *control* condition blamed the victim more (*M* = 2.05, *SD* = 0.94) compared with the participants in the *sexual objectification* condition (*M* = 1.42, *SD* = 0.62; LSD *p* < 0.001) and marginally more than participants in the *ostracism* condition (*M* = 1.72, *SD* = 0.88; LSD *p* = 0.056). Participants in the *ostracism* condition blamed the victim marginally more than participants in the *sexual objectification* condition (LSD *p* = 0.079).

To summarize, women who experienced sexual objectification attributed less blame to the victim (the woman) than women who were treated well. The extent to which ostracized women blamed the victim fell in between women who experienced sexual objectification and women in the control condition, and marginally differed from both. Thus, in study 2, we aimed to explore whether empathy can mediate the effect. If the degree to which one can empathize with another depends on the similarity of their own situation to that of the target, then sexually objectified women should empathize with a victim of sexual harassment the most, which will in turn reduce victim blaming. Because ostracism and sexual objectification share similarities, ostracized women may be able to empathize with the target of sexual harassment as well, more so than women who are treated well, but less so than women who were sexually objectified themselves. In addition, in study 2, we chose to utilize a different stimulus to examine the generalizability of the effect. Instead of the newspaper article that dealt with a relatable young woman who was attempting to study for an exam with a male friend, we used a video clip in which a well-known talk show host is interviewing an actress.

## Study 2

### Methods

#### Participants and design

In total, 181 women (*M*_*age*_ = 23.24, *SD* = 2.85) participated in the study virtually from their personal computers. An *a-priori* power analysis to achieve 80% power based on the effect size of study 1 (α = 0.05; partial η^2^ = 0.1) determined a desired sample size of 144 participants to reveal a significant effect for ANOVA, and 120 for the mediation analysis (α = 0.05; effect size *f*^2^ = 0.15). We recruited additional 25% to account for possible attrition. Participants were recruited using an internet-based platform called iPanel. Relevant participants who take a part in this online panel were invited to participate in the study. Exclusion criteria were participants with prior experiences of sexual assault. Participants were randomly assigned to one of three conditions: ostracism, sexual objectification, and control.

#### Procedure

The procedure was similar to the procedure in study 1. Participants underwent the same manipulation as in study 1 and were either ostracized, sexually objectified, or treated well by a video-taped man. After watching the video, participants described what they mentally visualized during the exercise, and completed objectification-related questionnaires.

Then, the participants watched a video^2^ in which a talk show host (Jay Leno) interviewed an actress (Judith Light). During the interview, the interviewer insisted on talking with the actress about sex, asking provocative questions and touching her in a sexual manner. He continued to do so even after the actress was visibly uncomfortable and tried to change the subject multiple times. After watching the interview, participants completed the same measures as in study 1 including event interpretation and attribution of blame.

At last, participants completed manipulation and attention checks, indicated whether they encountered technical issues during the study, and completed a demographic background questionnaire.

#### Measures

Measures were identical to the measures in study 1: sexual objectification (α = 0.85), self-objectification (α = 0.73), and victim blaming (α = 0.91); with the addition of the following measures. All the measures were on a 7-point scale ranging from not at *all* (1) to *extremely* (7).

##### Manipulation and attention checks

###### Subject of video

Participants were asked to indicate the subject of the video: meeting between business partners, a talk show host (man) interviewing an actress, a talk show host (woman) interviewing an actor, or instructions video for a device.

###### Empathy

Participants were asked to indicate the extent to which they felt empathy toward the woman (“To what extent do you empathize with the woman in the video?”; 1 item).

#### Statistical analysis

To examine the effects of the condition (i.e., ostracism, sexual objectification, and control) on the outcome variables a series of one-way ANOVA was conducted, unless stated otherwise.

### Results and discussion

### Preliminary analysis

#### Manipulation checks

##### Eye gaze direction

A chi-square test of independence was performed to examine the relation between the video conditions and the participant's perception of the man's eye gaze direction. The relation was significant, $\chi_{\left(4\right)}^{2}$ = 129.85, *p* < 0.001, indicating that the majority of the participants in each condition correctly identified the direction of the man's eye gaze: ostracism (side eye gaze; 70.9%), sexual objectification (down eye gaze; 65.6%), and control (direct eye gaze; 82.5%).

##### Subject of video

All the participants reported watching a video presenting a talk show host (man) interviewing an actress.

#### Process check

Participants in the *sexual objectification* condition felt more sexually objectified as compared with both the *control* and *ostracism* conditions, *F*_(2, 178)_ = 19.20, *p* < 0.001, *partial* η^2^ = 0.18 (LSD simple effects < 0.001). In addition, participants in the *sexual objectification* condition reported higher self-objectification as compared with the *control* condition, *F*_(2, 178)_ = 4.65, *p* = 0.011, *partial* η^2^= 0.05 (LSD simple effect *p* = 0.003). For means and SDs see Table 1.

### Main analysis

#### Empathy for the woman

Participants in the *sexual objectification* condition empathized with the woman as compared with both the *control* and *ostracism* conditions, *F*_(2, 178)_ = 3.50, *p* = 0.03, *partial* η^2^ = 0.04 (LSD simple effects *p* < 0.03). For means and SDs see Table 2.

**Table 2:** Means and standard deviations of empathy for the woman in the video and victim blame as a function of manipulation condition (face, away, body).

	**Face**	**Away**	**Body**
Empathy for the woman	4.11 ± 2.05	4.16 ± 2.10	4.93 ± 1.75
Victim blame	4.16 ± 1.82	4.21 ± 0.82	3.94 ± 0.75

#### Victim blame

No significant effect was found to eye gaze direction manipulation on victim blaming, *F*_(2, 178)_ < 1. For means and SDs see Table 2.

#### Mediation analysis

Because the lack of a direct effect does not rule out the possibility of a significant indirect effect, a mediation analysis was conducted to examine whether sexual objectification and ostracism lead to greater empathy toward the victim, which in turn leads to lower victim blaming. For this analysis, a mediation model with a three-level categorical independent variable (i.e., condition: *face, body*, and *away*) was conducted using model 4 in the PROCESS macro for SPSS (Hayes, 2013). A bootstrapping procedure of 10,000 resamples was used to generate 95% CIs around the coefficients, and the direct and indirect effects for inference testing. Ninety-five percent CIs not containing zero indicate a significant effect.

**Figure 1:**
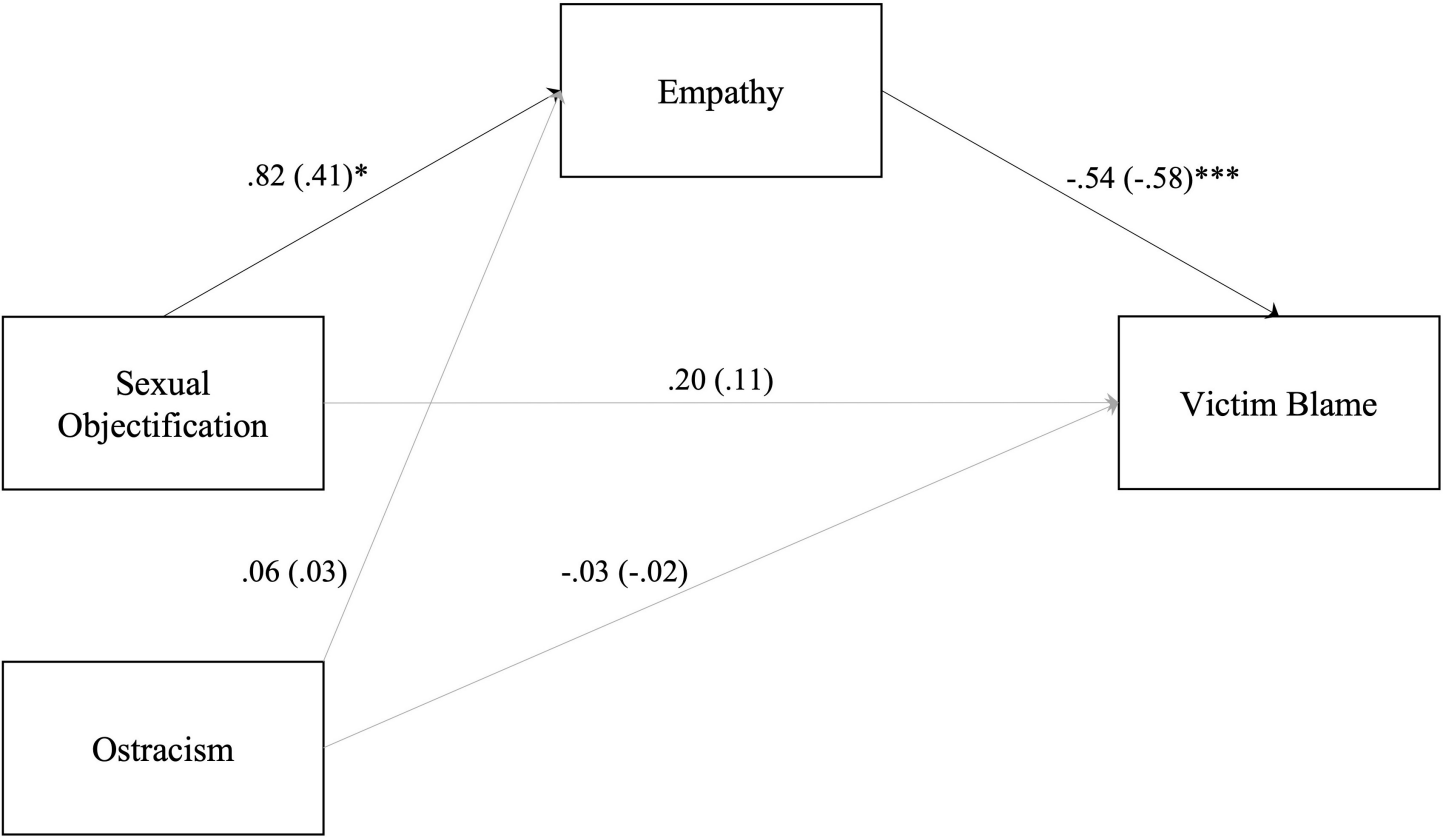
Mediation model presenting the mediating role of empathy in the effects of sexual objectification (body condition) vs. neutral (face condition) and ostracism (away condition) vs. neutral on victim blame. *p < 0.05, ***p < 0.001.

As seen in Figure 1, participants in the *sexual objectification (body)* condition (vs. *control-face* condition) reported greater empathy toward the woman. The extent to which they felt empathy was significantly associated with lower victim blaming. This resulted in a significant indirect effect of condition on victim blaming through empathy [indirect effect = −0.24, 95% CI (−0.45, −0.04)]. The degree to which participants in the *ostracism (away)* condition felt empathy toward the victim did not significantly differ from those in the *control (face)* condition, thus, the overall indirect effect was not significant [indirect effect = −0.02, 95% CI (−0.25, 0.21)].

## General discussion

The findings from both studies are consistent in showing that an experience of sexual objectification reduces the tendency to blame another victim of sexual objectification. In study 1, women who were sexually objectified blamed the victim to a lesser extent than women who were treated well, with women who experienced ostracism falling in between: blaming the victim marginally more than sexually objectified women, and marginally less than women who were treated well. This is the first study to show effects of sexual objectification on victim blaming.

Study 2 was purposefully designed to impose a more challenging test of the effect. The scenario presented in study 2 was less relatable to the participants as it seemed more remote: the dynamic was between two famous individuals in Hollywood, and the interview took place several years ago—when norms regarding sexual misconduct were vague. Signals of approval and appropriateness of the treatment were communicated by the characteristics of the situation, including high-authority figure (the host), laughing audience, and the actress's outfit; all communicate that this is the norm of a talk show interview and resemble characteristics that were found to increase victim blame in previous research (Loughnan et al., 2013). In this study, a direct effect of sexual objectification or ostracism on victim blaming was not detected. This inconsistency of results may be due to the added complexity we stated earlier, additional evidence is needed to clarify the relationship between objectification and victim blaming. However, and more importantly, an indirect effect through empathy was detected: sexually objectified women experienced more empathy toward the victim, which was associated with reduced victim blaming. This finding alone raise questions regarding the way female victims are portrayed by the media (newspapers, TV, and social networks), and emphasize the need to present victims in a relatable manner to induce empathy and avoid victim blaming.

This work is first to demonstrate that the tendency to blame the victim is affected by situational factors of the perceiver. Whereas, research concerning the phenomena of victim blaming focused on characteristics of the victim, perpetrator, their relation, and the situation they are in, or on individual characteristics of the perceiver (e.g., rape myth acceptance; Bevens et al., 2018); we were able to demonstrate that experiencing sexual objectification (in both studies) and ostracism (in study 1) affected the tendency to blame the victim directly (study 1) and indirectly (study 2). It is known that the vast majority of adult women had experienced some form of sexual objectification (Bartky, 1990), and that all adult individuals (men and women) had experienced ostracism (Williams, 2009; Nezlek et al., 2012). In light of this, it seems that it is not simply the past experience that affects victim blame, but rather the immediacy and salience of that experience (manipulation during our studies) that affect the ability to empathize with the victim and in turn, the attribution of blame. Future studies should examine the interplay between situational factors and individual characteristics in the context of victim blame.

Previous research demonstrated that the more people perceive others as similar to themselves, the better they can sympathize and empathize with them (Davis, 1994; Batson et al., 2005). In a related vein, we propose that the more people perceive the situation others are in as similar to their own, the better they can relate and empathize with them—and that, in the context of sexual harassment reduces attribution of blame.

Sexual objectification is experienced as a form of ostracism. However, sexual objectification is a unique form of ostracism in that women still receive attention, although usually unwarranted, to their body and sexual functions while their core is being ignored. That makes sexual objectification resemble the sexual harassment in the studies the most, ostracism share some commonalities with sexual harassment but to a lesser extent and being treated well the least similar to sexual harassment. Thus, women who experienced sexual objectification were able to experience the greatest empathy to the victim and attributed less blame. Future research should explore this matching hypothesis further and examine whether the type of ostracism one experiences elicits empathy to others who go through a similar experience. Further avenues for future research include examining whether the exposure to other sexist behaviors (e.g., verbal harassment or sexist humor) could also increase sense of common fate and empathy among women and provoke similar effect on victim blaming; whether trait self-objectification leads to a similar empathy–victim blaming relationship and how it may interact with state sexual objectification; and at last, examine different gender compositions.

In our studies, we have examined empathy using a single item asking directly regarding feelings of empathy. Still, empathy is a complex phenomenon, with investigators referring to two aspects. Cognitive empathy relates to the cognitive nature of empathy, emphasizing the ability to adopt another person's point of view (perspective taking) and theory of mind (e.g., see Davis, 1994; Eslinger, 1998). Emotional empathy relates to the emotional facets of empathy. Referring to one's affective reactions to the experience of others (Davis, 1994) and to aspects of helping behavior (Batson et al., 1981). The main difference between emotional and cognitive empathy is that the latter relies on cognitive and intellectual understanding of another person's point of view that is a slow and high process, whereas the former adds sharing of another person's feelings, which is elicited immediately and automatically (Mehrabian and Epstein, 1972; Fuchs, 2017). In our studies, we did not differentiate between types of empathy. Since we manipulated similar situations of the perceiver and the sexually harassed target one can hypothesize that automatic emotional aspect of empathy was at play (congruent feelings of perceiver and target). Unfortunately, we were unable to test this in the current research. Future examination of different aspects of empathy in the context of objectification, ostracism, and victim blaming is warranted.

## Data availability statement

The raw data supporting the conclusions of this article will be made available by the authors, without undue reservation.

## Ethics statement

The studies involving human participants were reviewed and approved by Max Stern Yezreel Valley College IRB. Participants indicated their informed consent electronically.

## Author contributions

MD and MN involved in all stages of research including planning and executing the research, analyzing the data, and crafting the manuscript. Both authors contributed to the article and approved the submitted version.

## Funding

This research was conducted with the support of the Center for Integration in Science at Ministry of Aliya and Integration, State of Israel.

## Conflict of interest

The authors declare that the research was conducted in the absence of any commercial or financial relationships that could be construed as a potential conflict of interest.

## Publisher's note

All claims expressed in this article are solely those of the authors and do not necessarily represent those of their affiliated organizations, or those of the publisher, the editors and the reviewers. Any product that may be evaluated in this article, or claim that may be made by its manufacturer, is not guaranteed or endorsed by the publisher.

## Footnotes

^1^Relevant measures are presented below, we have collected additional measures including self-esteem for other purposes.

^2^Link to the video: https://youtu.be/ntyA18mRMHk.
