# Supplementary figures and images for: Would victims blame victims? Effects of ostracism, sexual objectification, and empathy on victim blaming
Source: Front Psychol. 2022 Aug 1;13:912698. doi: 10.3389/fpsyg.2022.912698 (PMC9376598; doi:10.3389/fpsyg.2022.912698)

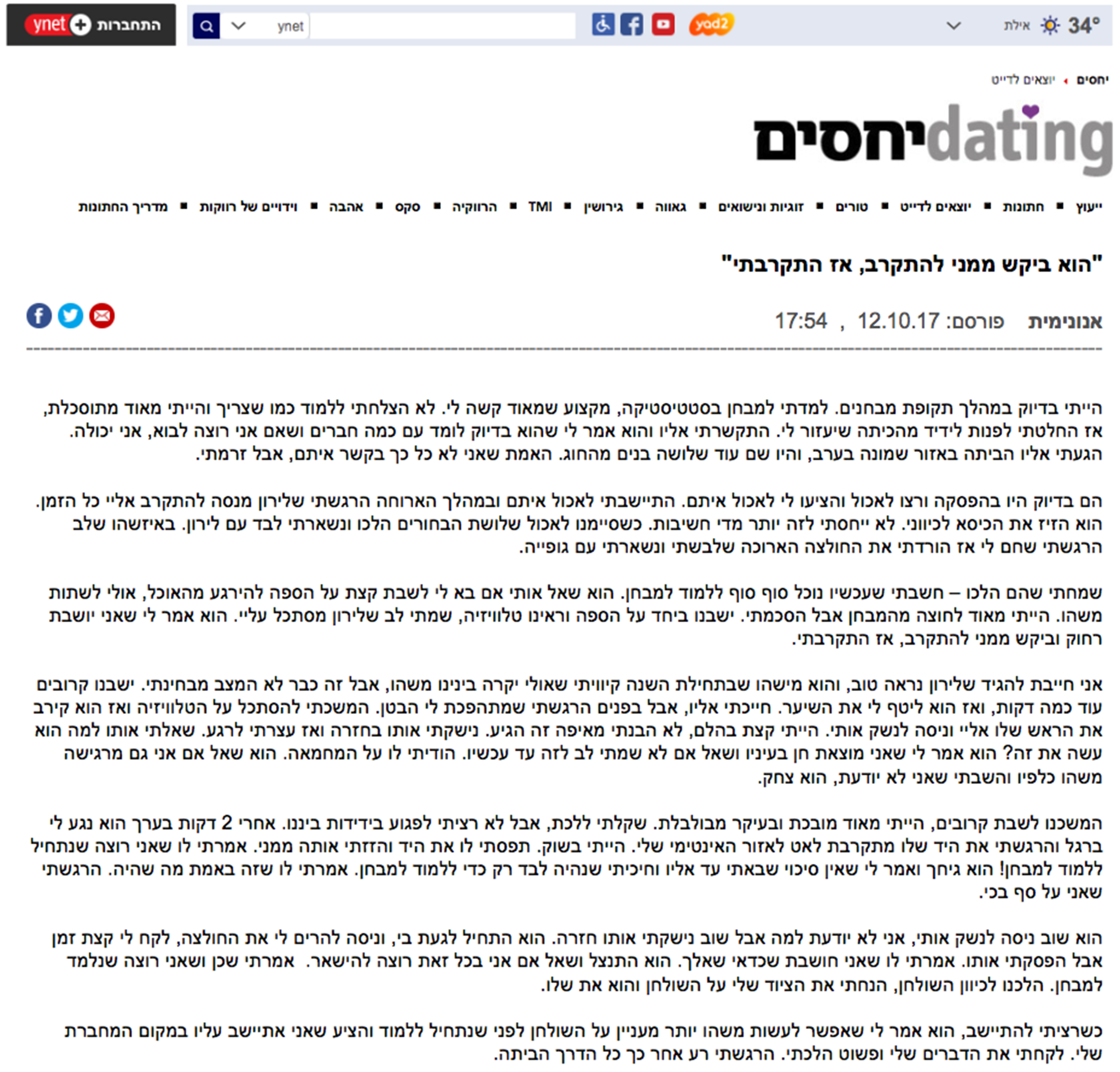

Supplement: Supplementary file 1 [file Image_1.png]

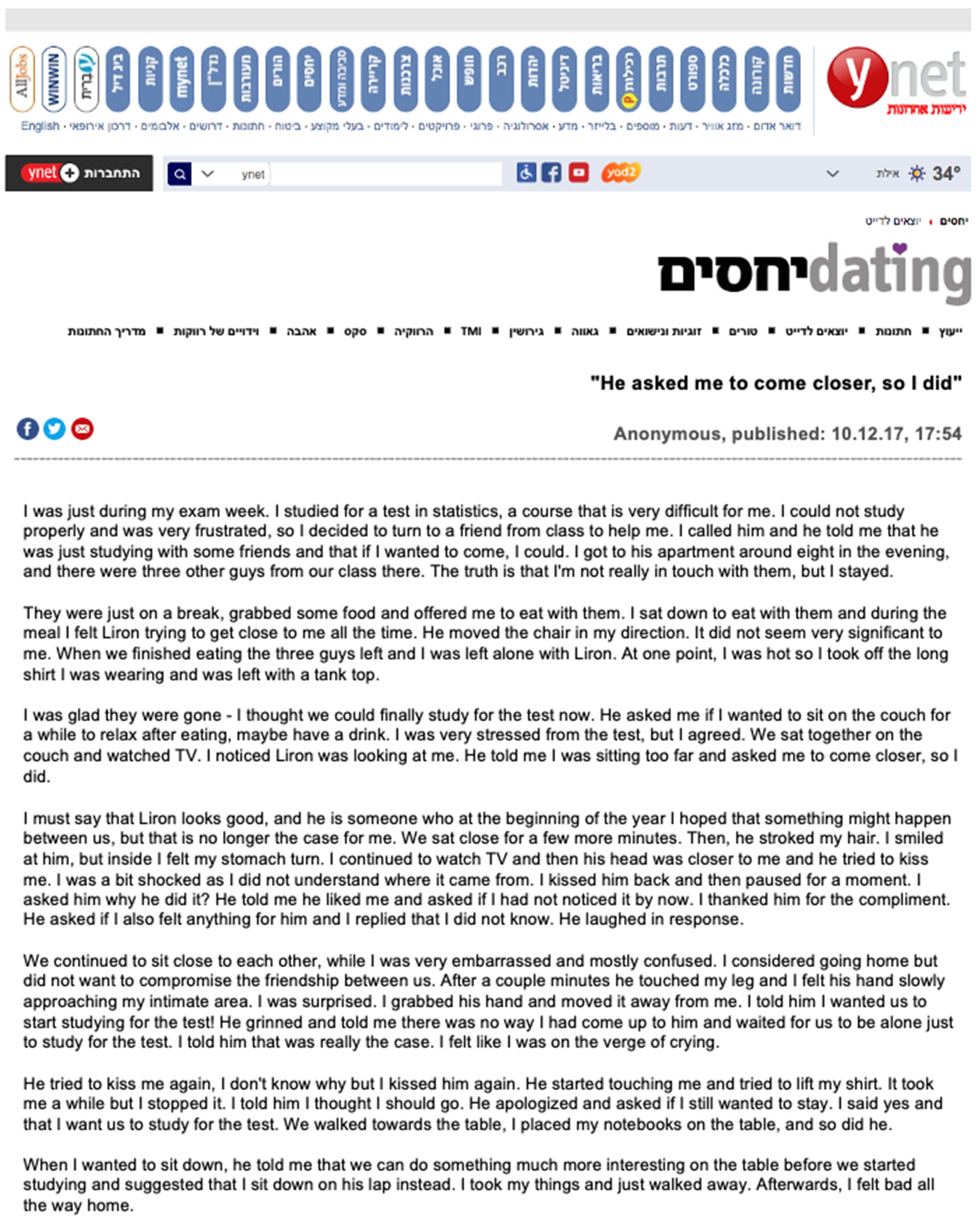

Supplement: Supplementary file 2 [file Image_2.png]
